# Modified rubber band ligation for treatment of grade II/III hemorrhoids: clinical efficacy and safety evaluation—a retrospective study

**DOI:** 10.1186/s12893-022-01688-8

**Published:** 2022-06-20

**Authors:** Jiazi Yu, Jie Zhong, Tao Peng, Liangbin Jin, Leibin Shen, Mian Yang

**Affiliations:** 1 Department of General Sugury, Ningbo Medical Treatment Centre Li Huili Hospital, 1111 Jiangnan Road, Ningbo, 315000 People’s Republic of China; 2 Li Huili Hospital of Ningbo University, Ningbo, 315000 People’s Republic of China; 3 Department of General Surgery, Qianhu Hospital, Ningbo, 315020 China

**Keywords:** Hemorrhoids, Rubber band ligation, complications, Curative effect

## Abstract

**Background:**

Massive, delayed bleeding (DB) is the most common major complication of Rubber Band Ligation (RBL) for internal hemorrhoids caused by premature band slippage. In this study we modified conventional RBL to prevent early rubber band slippage and evaluated its clinical efficacy and safety.

**Methods:**

Study participants were consecutive patients with grade II or III internal hemorrhoids treated with RBL at Ningbo Medical Center of Lihuili Hospital from January 2019 to December 2020. Postoperative minor complications such as pain, swelling, anal edema, prolapse recurrence and major complications like DB were retrospectively reviewed.

**Results:**

A total of 274 patients were enrolled, including 149 patients treated with modified RBL and 125 treated with conventional RBL. There was no statistically significant difference between the two groups at baseline. Five cases of postoperative DB have been observed in the conventional RBL group, compared to none in the modified ones, with a significant difference (P < 0.05). Within three months after surgery, 8 cases in the modified RBL group experienced a recurrence rate of 5.4%, whereas 17 patients in the conventional RBL group experienced a recurrence rate of 13.6%. The difference was statistically significant (P < 0.05). The VAS score, edema, and incidence of sensation of prolapse between the two groups were not significantly different at 3 and 7 days after surgery (P < 0.05). There were also no significant differences in HDSS and SHS scores between the two groups after surgery (P > 0.05).

**Conclusion:**

Modified RBL may be associated with a lower rate of complications, especially with lower DB rate in comparison with standard RBL. Further studies in larger samples and different design are necessary to confirm these results.

## Background

Hemorrhoidal disease (HD) is a common anal disorder and one of the most common findings observed in colorectal clinics [1, 2]. Approximately 50% of people present at least one episode of symptomatic hemorrhoids during their life, and majority of patients will undergo surgery if conservative treatment does not improve their condition [3, 4]. There have been many advances in the surgical approach to HD over the past century. The development of new surgical and office-based procedures has led to reduced postoperative pain and complications and improved long-term outcomes [4]. The American Society of Colon and Rectal Surgeons Clinical Practice Guidelines for the Management of Hemorrhoids recommended that for most patients with grade I and II and select patients with grade III internal hemorrhoidal disease who fail medical treatment, office-based procedures, including RBL, sclerotherapy, and infrared coagulation can be effective [5]. Over the last two decades, RBL has proven to be one of the most popular and effective treatments and superior to office-based procedures [6, 7]. Ligation of the hemorrhoidal tissue results in necrosis of the prolapsing mucosa, followed by scar attachment to the rectal wall [8]. As the ligature is placed above the dentate line, where somatic sensitivity is absent, this quick technique is well tolerated by patients, and they can get back to daily life sooner [9].

However, RBL is not free from complications [10]. Minor complications include band slippage, pain, or bleeding, which are usually self-limiting. In contrast, major complications, including DB, severe thrombosis, mucosal ulcers, prostatic abscesses, etc., are rare. However, these may be life-threating [11]. DB, regarded as almost arterial and projectile with hypovolemic shock, appears to be the most common among these complications. It usually occurs days after the procedure and is related to the resultant ulcer [10]. Premature band slippage may be the key risk factor for ulceration because slippage may lead to incomplete necrosis of tissue, which may lead to ulcer-induced DB, as well as hemorrhoids prolapse recurrence [12–14].

Since January 2020, our center has improved the RBL procedure to prevent premature rubber band slippage and reduce DB and prolapse recurrence rates. The improved RBL procedure is what we call this modified RBL. This investigation aims to determine the efficacy and safety of modified RBL compared to conventional RBL.

## Methods

### Patients

The study was a retrospective analysis based on STROBE guidelines (Strengthening the Reporting of Observational Studies in Epidemiology (STROBE) statement for cohort studies [15]. The Ethics Committee of the Ningbo medical center Lihuili hospital approved the study. Patients who underwent surgery for internal hemorrhoids between January 2019 and December 2020 at Ningbo Medical Center Lihuili Hospital were retrospectively reviewed. Inclusion criteria were as follows:(1) a clinical diagnosis of grade II or III HD, based on Goligher’s classification [16]; (2) RBL surgery was performed; (3) age 18–75. Patients with anal fissures, anal fistulas, anal sinusitis, other perianal disorders, mental illness, or insufficient clinical and follow-up information were excluded. The patients treated with conventional RBL procedures from January to December 2019 were divided into the conventional RBL group; those treated with modified RBL procedures from January to December 2020 were divided into the modified RBL group. All participants were enrolled consecutively.

### Surgical methods

All patients received oral administration of compound polyethylene glycol for intestinal preparation in the afternoon of the day before the procedure. No antibiotics were administered. The same medical group completed the surgeries. 15.3% of procedures involved block anesthesia in a prone jackknife position, and 84.7% were performed under spinal anesthesia. According to our experience, anesthesia is essential for gaining an optimal operating view to perform a correct ligation. In most cases, spinal anesthesia was preferred to achieve operating view, especially in the male, overweighted or muscular patients. We will selectively perform block anesthesia on female patients because it may be enough to gain the optimal operating field for the ligation. Patients with underlying conditions that require anticoagulants (such as cardiovascular disease) should be hospitalized. Five days prior to surgery, the patient should be switched to low molecular weight heparin and switched back to anticoagulants 24 h after surgery.

In the RBL group, the procedure was performed using an anuscope, which was inserted and placed between 1 and 2 cm above the dentate line. We routinely assessed the hemorrhoids' distribution, identified the symptomatic ones, and identified the sites of ligation. We treated all internal hemorrhoids that were symptomatic. The ligator was connected to a negative pressure suction device in the next step. The hemorrhoids were allowed to prolapse into the anuscopee lumen and then sucked into it. When the negative pressure reached 0.08–0.1 MPa, a rubber O-ring was ejected around the base of the hemorrhoids. One hemorrhoid was ligated in one session in 97 cases (35.4%), and two hemorrhoids in 159 patients (58.0%). Rubber bands were placed at different levels in the rectum to prevent rectal stenosis when the three main hemorrhoidal piles should be ligated in 1 session (18 cases, 6.6%).

**Fig. 1:**
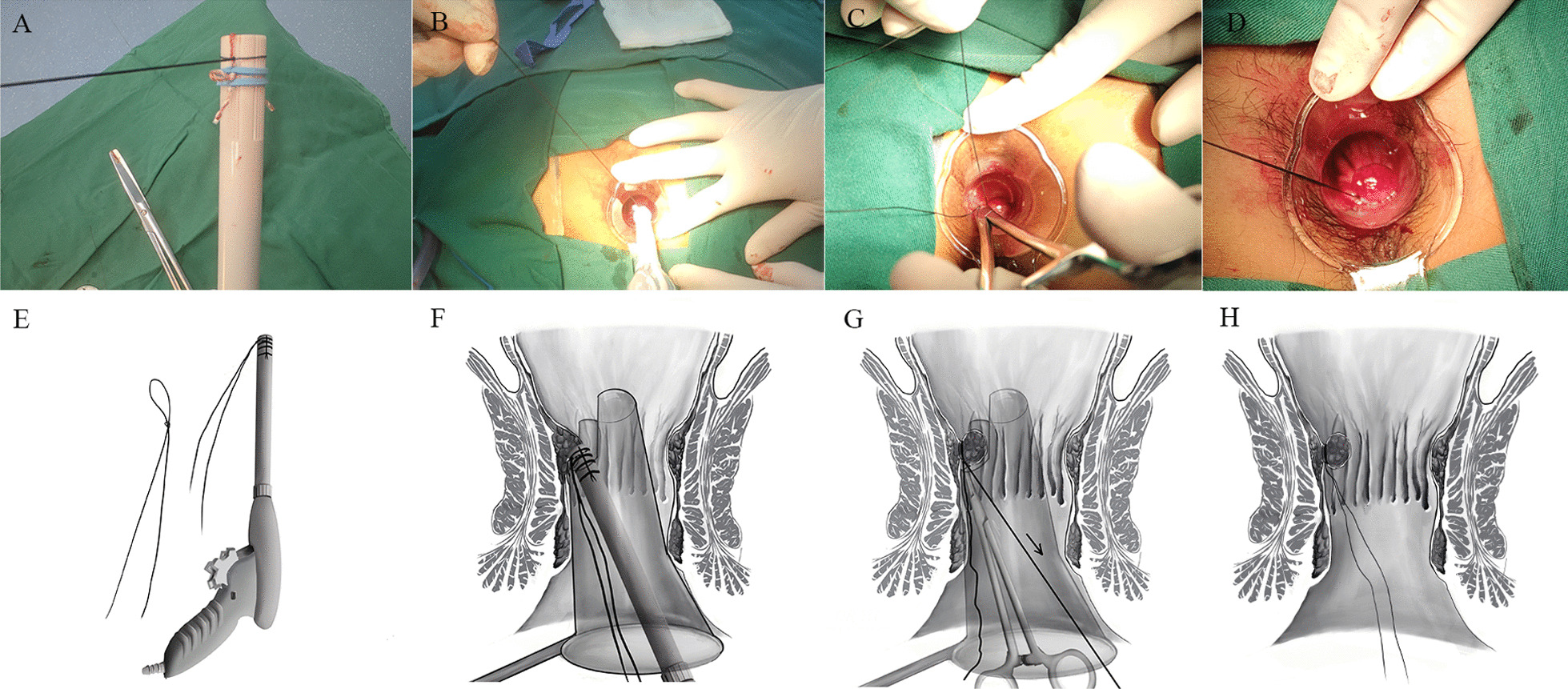
The RBL modified technique proposed by the authors. **A**, **E** A “fisherman's knot” was made with no. 4 silk wire, and set in front of the rubber ring. **B**, **F** Release the rubber ring and coil to the bottom of the tissue together. **C**, **G** Use the needle holder, hold one thread and keep the coil under the rubber ring and manually tighten the other line by hand. **D**, **H** Schematic of the final effect

The basic procedures in the modified RBL group were similar to those in the conventional RBL group with the following modified steps. Firstly, a "fisherman's knot" was made anterior to the rubber ring of the ligator to be excited using the 4–0 suture, thus releasing the coil and rubber ring together at the base of the hemorrhoids (Fig. 1A, B, E, F). Secondly, hold the thread with a needle holder to maintain the loose loop beneath the rubber band. Hold the other line while slowly tightening it. When drawing it, the tightness of the coil should be concerned, which was narrowed to hold the mucosal bulb in place, rather than the ligation (Fig. 1C, G). Finally, cut the silk thread (Fig. 1D, H). One ligation was performed in 96 patients (30.4%) of the BC group, while multiple ligations were performed in the remaining, including two ligations in 127 cases (40.2%) and three ligations in 93(29.4%).

### Observation indicators

Postoperative complications were recorded during follow-up. All patients were followed up at the first week and the fourth weeks at the clinic, and telephone follow-up was performed at third month and the end of the first year. The primary outcomes included the recurrence rate and incidence of DB. The secondary outcomes included the incidence of perioperative complications (e.g., the degree of pain, anal edema, and anal swelling). The Visual Analogue Scale (VAS) was used to assess the pain level: 0 indicates no pain, 1–3 indicates mild pain, 4–6 indicates moderate pain, and 7–10 indicates severe pain. Patients were self-graded for VAS according to their self-perception. The anal edge edema was postoperatively examined. Sensation of prolapse was recorded according to the postoperative self-perception of patients. Additionally, symptom severity and quality of life of patients were assessed using the Hemorrhoidal Disease Symptom Score (HDSS) and Short Health Scale for HD (SHS-HD) at enrollment and the 3rd and 12th months thereafter. HDSS measures pain, itching, bleeding, soiling and prolapse on a 5-point scale (0 = never, 1 = less than once a month, 2 = less than once a week, 3 = 1–6 days per week, 4 = every day or always). The SHS-HD score includes 4 questions, each with a 7-point Likert scale (1 is the minimum score, 7 is the maximum score) [17].

### Statistical analysis

For categorical variables, data is presented as frequency with percentage and mean ± standard deviation for continuous variables. As appropriate, we used chi-square tests or Fisher Exact Tests to compare categorical variables across subgroups and independent tests for continuous variables. The statistical analyses were conducted using IBM SPSS Statistics ver. 22.0 (IBM Corp., Armonk, NY, USA).

## Results

### Basic information of enrolled patients

A total of 274 patients were enrolled, including 149 patients treated with modified RBL and 125 treated with conventional RBL. Fifty-six patients missed their clinic follow-up, so we contacted them by phone and inquired about their postoperative recovery. There were no missed visits, and the longest follow-up was 12 months. There were no statistically significant differences between the two groups in age, sex, BMI, hemorrhoid grade, history of hemorrhoid surgery, constipation, NO. of ligated sites, main symptoms, cause of treatment, use of anticoagulants, HDSS score and SHS score at baseline (P > 0.05). As shown in Table 1.

**Table 1:** Information about the patients involved in the study

The general information	Modified RBL group (149)	Conventional RBL group (125)	Statistics	P values
Age (years)	38.5 ± 8.9	39.6 ± 10.3	0.95	0.34
Gender				
Male	65	51	0.22	0.71
Female	84	74		
BMI	22.8 ± 3.9	23.2 ± 4.3	0.81	0.42
Hemorrhoid grade				
Grade II	56	41	0.68	0.48
Grade III	93	84		
Previous history of hemorrhoid surgery				
There are	7	4	0.40	0.76
There is no	142	121		
Constipation				
Yes	12	8	0.27	0.65
No	137	117		
No. of ligated sites				
1	50	47	0.68	0.41
2	88	71		
3	11	7		
Main symptom and cause of treatment				
Bleeding	58	43	1.57	0.46
Prolapse	70	68		
Pain	21	14		
Anticoagulant				
Yes	7	3	1.02	0.35
No	142	122		
HDSS	10.9 ± 0.8	11.0 ± 0.7	1.09	0.28
SHS	18.3 ± 3.2	18.1 ± 2.9	0.54	0.59

### Major complications

Neither group experienced postoperative complications such as hematomas, allergies, arrhythmias, anal fistulas or rectal stenosis. There was no case of DB in the observation group, while there were 5 cases of DB in the control group, accompanied by a significant progressive decrease in hemoglobin. All the bleeding patients were cured by anal suture hemostasis without blood transfusion, and there was a significant statistical difference between the two groups (P < 0.05). In comparing recurrence rate within three months after surgery, 8 cases in the observation group had a recurrence rate of 5.4%, while 17 patients in the control group had a recurrence rate of 13.6%. The difference was statistically significant (P < 0.05). Subgroup analysis showed 1 case of recurrence in the observation group of grade II hemorrhoid and 2 cases in the control group. There was no statistical difference between the two groups (P > 0.05). There were 7 cases of recurrence in the observation group and 15 cases in the control group, resulting in a recurrence rate of 7.5% and 17.9%, respectively. Difference between the two groups was statistically significant (P < 0.05, shown in Table 2).

**Table 2:** Incidence of postoperative complications

The evaluation index	Modified RBL group (149)	Conventional RBL group (125)	Statistics	P values
Total recurrence (cases/%)	8 (5.4)	17 (13.6)	5.55	0.02
Grade II recurrent hemorrhoids (cases/%)	1 (1.8)	2 (2.4)	0.76	0.39
Grade III hemorrhoid recurrence (cases/%)	7 (7.5)	15 (17.9)	4.33	0.04
Massive bleeding (cases/%)	0	5	-	0.02
Postoperative pain VAS				
After 3 days	3.5 ± 2.7	3.1 ± 3.3	0.27	0.14
7 days after	1.4 ± 1.2	1.3 ± 1.4	0.52	0.26
Anal edema (cases/%)				
After 3 days	33 (22.1)	39 (26.9)	0.90	0.34
7 days after	11 (7.4)	11 (8.8)	0.19	0.67
Sensation of prolaps (cases /%)				
After 3 days	34 (22.8)	26 (20.8)	0.16	0.69
7 days after	13 (8.7)	9 (7.2)	0.22	0.64
Discharged within 24 h	92 (61.7)	37 (29.6)	28.20	< 0.01
HDSS at the third month	1.8 ± 1.1	1.9 ± 1.4	0.67	0.51
HDSS at the 12th month	1.7 ± 0.8	1.8 ± 1.1	0.87	0.39
SHS at the third month	5.9 ± 1.7	6.2 ± 1.9	1.37	0.17
SHS at the 12th month	5.7 ± 1.0	5.9 ± 1.9	1.06	0.29

### Minor complications

There were different degrees of pain, edema, and sensation of prolapse in both groups three days after surgery, and there was no statistical difference in these complications between the two groups (P > 0.05) 0.7 days after surgery, the above conditions were alleviated in both groups, and there was no statistical difference in these complications between the two groups (P > 0.05).

Although HDSS and SHS scores of both groups decreased significantly 3 and 12 months after surgery, there were still no significant differences in the scores between the two groups (P > 0.05), which was possibly attributed to prompt management of complications (bleeding, prolapse) once they occurred.

## Discussion

Although the newest techniques are available in clinical practice, RBL remains the most effective method to treat second- and third- grade HD, especially in cases of bleeding and prolapsing hemorrhoids, with a high level of evidence [5, 18–20]. In comparison to other office-based procedures, sclerotherapy serves increasingly as a solid alternative therapy for grade I–II hemorrhoids [21–23]. Moser et al. [22] and Gallo et al. [23] reported that sclerotherapy with 3% polidocanol foam was a safe, effective, repeatable and low-cost procedure instrumental in the treatment of bleeding hemorrhoids. A randomized controlled study by Salgueiro et al. [21] suggested that compared with RBL, sclerotherapy with polidocanol foam had higher effectiveness, a shorter course of treatment, a lower recurrence rate, and fewer post-operative complications. A possible explanation is that the premature slippage of the rubber ring arising from conventional RBLs worsens the effect and induces more complications. In a rubber band ligation, the ligator is inserted through the anuscope to grasp or suction hemorrhoids to place the rubber band over it. As hemorrhoids are necrotic, a virtual mucopexy results when the anal mucosa is pulled upward by the necrotic base, puckering the mucosa together, thus elevating the inferior mucosa [24, 25]. Rubber rings have a large pore size and are highly prone to aging, resulting in reduced elasticity due to the rubber material. Consequently, rubber rings tend to slip prematurely after surgery, resulting in incomplete tissue necrosis and excessive ulcer surface after shedding. DB and surgical failure commonly result from this condition [26]. A common cause of treatment failure is hemorrhoids prolapse recurrence after surgery. Prolapse recurrence rates vary, with 6.6% to 18% of patients undergoing RBL requiring additional treatment sessions due to recurrent symptoms [27]. In this study, 9% of patients had recurrence within three months, requiring surgical treatment again. DB after RBL typically occurs after 10–14 days, at a rate of 1.2% to 2.5% [12, 28]. Five patients in this study had DB on the 5th to 7th day after surgery in the conventional RBL group. A progressive drop in hemoglobin and definite arterial bleeding was observed, which was stopped by surgical ligation. It is therefore urgent to improve the conventional RBL. 

Pata et al. [29] and Kang et al. [30] recently modified the RBL procedure, in order to prevent premature rubber band slippage, and reduce some typical complications of the procedure. Pata et al. combined rubber band ligation with 3% POLIDOCANOL foam sclerotherapy (sclerobanding) to treat second- and third-GRADE HD. Kang et al. used a nonabsorbable polymer ligature clip, which is 10% longer than conventional ligature clips, as an alternative to a rubber band. Comparing these modifications with conventional RBL leads to a significant reduction in postoperative complications. We attempted other methods, such as double rubber band ligation and 50% glucose or polyglactin injection into the mucosal bulb. However, the results have not been satisfactory. Since January of 2021, the modified RBL procedure described here has treated Grade III hemorrhoids. Based on the findings of this study, compared with the conventional RBL procedure, no complications of massive DB occurred after the procedure improved, and the recurrence rate of hemorrhoids prolapse significantly reduced after surgery (P < 0.05). Neither the traditional RBL procedure nor the modified RBL procedure showed significant differences in minor complications such as pain, swelling, or edema (P > 0.05). Compared to Pata et al. and Kang et al. our modification is simpler and more economical. In our opinion, the modified RBL procedure has the following advantages: A ligated mucosa is often not large enough. When a person strains to defecate, the mucosal bulb below the collar is retained by a silk thread, which prevents the collar from falling off prematurely. The silk wire of No. 4 has high strength, high friction, extremely stable fixation, medium thickness, does not form linear cutting, does not increase the patient's sense of foreign bodies, and does not increase the cost of treatment. The silk wire of No. 4 is not completely tightened, so the silk wire only serves to fix the mucous membrane ball and retains the advantages of the rubber ring ligation, such as slow elastic ligation. After the ligation tissue has fallen off, the ulcer surface is small, and the hemorrhagic blood vessel is completely occluded, eliminating the risk of postoperative bleeding. Additionally, 61.7% of modified RBL group patients were admitted to the day-care unit and discharged within 24 h, compared with 23.4% of the convention RBL group. Despite this, the modified RBL procedure is not associated with increased postoperative complications. The results of this study indicate that the modified RBL procedure is also safe and effective in the day-care unit.

The modified RBL has improved the effectiveness and reduced the complications, but it is still not as convenient as sclerotherapy. Hence, sclerotherapy may be better suited to treat patients with grade I/II internal bleeding HD than the RBL. Nevertheless, for patients with hemorrhoids complicated by prolapse or characterized mainly by prolapse, RBLs may be a better option since sclerotherapy cannot pull the prolapsed tissue upward. More importantly, RBLs and sclerotherapy can be combined in the treatment of HD. The combination can maximize each procedure's strengths and broaden the indication for the treatment of anal disorders such as grade IV hemorrhoids, rectal mucosal prolapse, and rectovaginal prolapse. However, this view requires further validation of clinical studies.

## Conclusion

The results of this study demonstrate that a modified RBL procedure can significantly reduce the postoperative recurrence rate of grade III hemorrhoids, improve the effectiveness of treatment, and considerably reduce the occurrence of severe complications of massive postoperative bleeding. Furthermore, the modified RBL procedure is simple to perform, does not increase the incidence of minor postoperative complications, does not increase the surgery cost, and can be carried out on the outpatient or day surgery unit.

The study has some limitations. Firstly, all procedures are performed by the same colorectal surgeon, so all potential biases are inherent to this study design. Moreover, no comparison with other treatments has been conducted, and long-term results are lacking. It is necessary to conduct further studies comparing RBL with other therapies to determine the best treatment plan for reducing complications after surgery. Although 61.7% of modified RBL group patients were admitted to the day-care unit in this study, the proposed technique requires spinal/regional anesthesia and admission in the hospital, with increased costs and resources. As one of these office-based procedures, RBL should be used in clinic with no anesthesia or regional anesthesia. We will further evaluate the feasibility of performing this procedure without anesthesia or with regional anesthesia.

## Data Availability

Data used and/or analysed in the current study are available upon reasonable request from the corresponding author.

## Abbreviations

DB: Delayed bleeding
RBL: Rubber Band Ligation
BMI: Body mass index
STROBE: Strengthening the Reporting of Observational Studies in Epidemiology
VAS: Visual Analogue Scale
